# Supplementary material for: Association between Ambient Air Pollution and MRI-Defined Brain Infarcts in Health Examinations in China
Source: Int J Environ Res Public Health. 2021 Apr 19;18(8):4325. doi: 10.3390/ijerph18084325 (PMC8072670; doi:10.3390/ijerph18084325)
Supplement: Supplementary file 1 [file ijerph-18-04325-s001.zip › ijerph-1137970-supplementary.pdf]

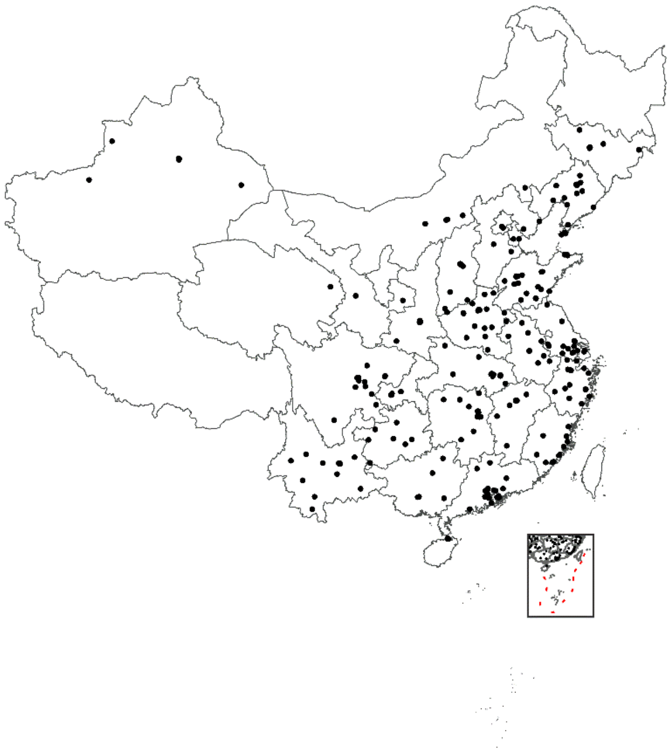

Supplementary Figure 1. Map of 174 cities from 30 provinces of China included in the study

Supplementary Table 1. The city-level characteristics in 174 cities in the study

Province/autonomous regions/municipalities	City	N	Age	$PM_{2.5}$ (μg/m^3)	PM_{10} (μg/m^3)	NO_2 (μg/m^3)	CO (μg/m^3)	Temperature (˚C)	Relative humidity (%)
Anhui	Bangbu	11930	45.8	61.2	94.5	36.8	0.897	16.1	79.3
	Chuzhou	1786	47.5	58.5	85.6	35.9	0.872	16.2	77.1
	Fuyang	609	43.1	59.9	90.6	36.1	0.854	16.3	76.4
	Hefei	19611	47.0	58.8	88.6	41.8	0.964	17.1	75.6
	Suzhou	2671	45.2	65.7	91.4	35.9	0.919	16.0	78.1
	Tongling	6324	47.4	55.3	85.7	42.4	1.215	17.7	79.7
	Wuhu	3377	47.1	52.3	81.5	41.3	1.006	17.0	76.1
	Xuancheng	1709	46.9	49.6	74.9	34.1	0.834	17.0	76.1
	Bozhou	11364	46.1	60.7	93.9	34.5	1.041	16.1	74.4
Beijing	Beijing	6341	46.1	68.8	97.9	45.7	1.112	11.7	57.9
Chongqing	Chongqing	12203	46.0	50.1	77	43.8	0.995	19.3	75.1
Fujian	Fuzhou	10775	47.0	26.6	51.3	27.6	0.69	20.8	78.3
	Longyan	2051	46.3	25.3	44	23	0.815	21.6	74.9
	Ningde	2207	47.2	26.9	45	20.7	0.848	20.7	77.1
	Putian	9390	42.6	28.2	44.3	18.2	0.657	20.8	79.5
	Quanzhou	3941	45.1	26	49.4	23.1	0.62	21.4	77.6
	Sanming	2283	45.7	27.1	46.3	24.5	1.125	18.2	82.3
	Xiamen	8766	41.4	27.5	46.7	26.6	0.562	21.4	77.6
	Zhangzhou	9774	43.9	33.8	61	29	0.707	21.6	74.9
Gansu	Jiuquan	377	43.9	41.4	127.3	29.8	0.658	7.9	46.2
	Lanzhou	18199	45.3	48.8	120.9	48.4	1.243	8.0	60.0
	Pingliang	4	50.8	40.6	87.6	40.8	1.002	9.3	63.2
	Qingyang	8812	45.8	35.4	75.2	18.4	1.043	10.1	58.7
Guangzdong	Dongwan	13236	42.1	36	51.5	36	0.856	22.7	79.4
	Foshan	6249	42.8	38.8	59.6	41.7	0.865	22.4	80.5
	Guangzhou	1826	44.2	35.7	56.6	45.6	0.923	22.2	81.3
	Heyuan	8685	45.5	31.8	48	23.8	0.881	22.5	75.0
	Huizhou	3691	44.2	27.5	49.1	23.1	0.797	22.5	75.0
	Jiangmen	7676	42.9	34.8	57.4	34.5	0.878	23.2	80.9
	Maoming	243	46.6	29.2	47.9	14	0.807	23.6	83.5
	Qingyuan	763	44.5	34.9	54.7	35.5	1.044	20.2	80.7
	Shaoguan	2426	44.6	34.8	52.3	26.2	1.014	20.7	81.5
	Shenzhen	21368	44.1	27.9	45.4	31.4	0.816	23.1	75.6
	Yangjiang	4610	45.3	31.7	46.9	18.9	0.955	23.0	83.2
	Zhaoqing	44	43.6	38.3	57.6	32.3	0.933	21.4	82.2
	Zhongshan	8752	43.6	31.2	46.6	30.3	0.921	23.1	75.6
	Zhuhai	10442	43.4	28.7	46.7	30.8	0.769	23.3	78.1
Guangxi	Guilin	2774	44.0	44.6	62.1	21.7	0.935	20.3	71.8
	Liuzhou	5237	45.5	45.6	68.3	24.3	1.051	19.9	77.7
	Nanning	17273	42.8	37	62.4	31.6	0.967	21.8	79.5

	Yulin	1488	46.7	36	53.5	20.1	1.06	22.4	81.7
Guizhou	Bijie	4855	45.5	30.8	47.2	23.5	0.981	13.6	79.9
	Guiyang	6052	43.0	34.7	57.6	25.6	0.706	14.9	80.0
	Liupanshui	3507	43.7	39.9	68.7	24.1	0.725	11.5	80.7
	Zunyi	4688	43.9	39.3	65.8	29.3	0.808	16.1	79.5
Hainan	Haikou	25078	47.6	20.2	37.3	12.6	0.622	24.7	86.1
Hebei	Baoding	6867	47.4	93.7	152.9	53.5	1.573	13.0	61.8
	Cangzhou	6397	48.2	67.9	113	44.7	1.156	13.7	62.5
	Qinhuangdao	2748	47.7	45.4	89.5	46.9	1.367	10.4	67.9
	Shijiazhuang	2	40.0	87.4	153.4	50.6	1.401	13.0	61.8
	Tangshan	2279	42.3	74.5	130	58.7	2.121	12.7	61.8
Henan	Anyang	53	48.7	85.5	146.9	48.1	2.029	16.1	59.0
	Hebi	3600	46.3	69.1	125.4	49.2	1.717	16.1	59.0
	Jiaozuo	921	51.3	82	139.7	43	1.679	16.1	59.0
	Kaifeng	9400	48.3	71	122.7	39.4	1.448	16.2	59.7
	Luoyang	11526	47.8	73.9	128.2	42.9	1.792	15.3	59.2
	Nanyang	1213	47.1	63.6	120.5	28.9	1.233	15.7	69.1
	Pingdingshan	9302	48.5	76.2	128.7	41.4	1.196	16.6	58.2
	Sanmenxia	2881	45.8	66.5	120.1	37.7	1.353	14.8	58.9
	Shangqiu	3371	46.2	71	121.6	33.7	0.855	14.8	73.9
	Xinyang	713	55.2	60.1	99.7	28.4	0.827	15.5	68.0
	Xuchang	1	31.0	70.4	121.6	45.7	1.408	16.2	59.7
	Zhengzhou	34865	46.2	81.2	146.4	53.3	1.407	16.3	58.7
	Zhoukou	3469	47.1	70	117	28.2	1.525	16.4	67.0
	Zhumadian	9546	47.5	66.4	116.9	35.8	0.933	16.4	67.0
	Luohe	5115	48.3	73.2	126.2	36.4	1.109	16.4	67.0
	Puyang	13063	49.5	72.9	131.9	40.6	1.517	16.2	59.7
Heilongjiang	Harbin	5	43.4	58.3	86.9	44.3	1.081	5.2	64.1
Hubei	E'zhou	2220	48.4	60.7	93.5	33.5	1.302	17.1	72.7
	Huanggang	2229	50.8	52.5	81.9	26.3	1.259	17.1	72.7
	Huangshi	5005	48.2	59.5	92.3	33	1.407	17.5	74.0
	Shiyan	10389	46.2	46.9	73.6	24.9	1.098	16.3	71.8
	Suizhou	1022	48.1	57	88.8	24.4	1.325	16.3	78.1
	Wuhan	42518	45.9	59	95.4	46.4	1.046	17.6	73.6
	Xianning	12	37.3	49.3	77.8	18.4	1.099	18.0	77.4
	Yichang	9011	45.7	63	99.4	34.6	1.063	16.8	76.7
Hunan	Changde	1156	47.1	50.7	77.5	19.8	1.196	17.9	80.7
	Changsha	30444	44.7	54.9	76.4	36.4	0.934	18.3	81.7
	Hengyang	10160	45.5	51.1	75.9	27.6	1.081	12.4	87.2
	Loudi	164	52.0	45	73.2	21.1	1.454	18.3	77.8
	Xiangtan	9027	44.0	53	86.2	34.4	1.007	18.3	81.7
	Yiyang	2526	47.7	45.6	84.9	25.5	1.022	18.6	76.3
	Yongzhou	5022	44.9	47.1	69.7	24	0.704	18.7	80.9
	Yueyang	5786	46.9	49.6	77.1	23.2	1.015	18.2	82.1

	Zhangjiajie	3785	46.6	43.9	71.5	17.9	1.52	16.9	80.3
	Zhuzhou	15783	45.6	51.2	81.2	32.2	0.831	18.7	78.9
Inner Mongolia	Baotou	10403	46.8	47.4	104	40.4	1.357	8.1	55.6
	Chifeng	2732	49.4	37	77.7	20.6	1.127	8.0	44.9
	Ordos	57	47.4	25.1	68.3	24.5	0.716	7.8	46.4
	Huhehot	21461	47.3	41.9	98.8	41.6	1.246	7.4	45.3
	Tongliao	20	54.6	42.7	82.1	22.2	0.671	8.3	50.8
	Ulanqab	855	52.0	35.5	66.6	29.6	0.666	4.4	50.2
Jilin	Changchun	26914	47.0	51.5	86.7	39	0.961	6.9	59.4
	Jilin	4661	49.8	48	78.4	29	0.872	6.2	64.7
	Songyuan	5463	47.7	39	76.8	23.2	0.955	5.8	62.5
Jiangsu	Changzhou	2807	44.9	50.1	84.9	44.3	1.015	17.5	70.8
	Lianyungang	941	47.2	45.2	79.4	29.2	0.846	15.4	73.0
	Nanjing	12419	46.0	48	86.5	46.7	1.022	17.0	73.3
	Nantong	6538	48.2	47.1	73.7	35.7	0.786	16.3	78.3
	Suzhou	28655	44.0	48.3	73.8	48.8	0.898	17.5	70.8
	Wuxi	6284	46.4	52.5	84.5	44.3	1.053	17.5	70.8
	Yancheng	2141	46.7	44.3	78.4	24.3	0.777	15.0	82.6
	Yixing	3483	52.6	45.9	66.3	35.3	1.031	17.5	70.8
Jiangxi	Fuzhou	465	42.7	42.6	62.1	18.3	1.094	19.0	78.4
	Ganzhou	9696	45.0	43.1	63.9	21.6	1.307	19.5	77.6
	Jingdezhen	385	49.3	39.9	64.4	15.8	0.756	19.1	76.2
	Nanchang	30417	45.6	40.7	75.4	31	0.983	19.2	73.0
	Shangrao	3361	47.6	41.4	67.2	27.6	0.857	18.9	75.6
	Yichun	6725	46.1	48	72	24.2	0.954	18.5	78.1
Liaoning	Anshan	97	50.7	54.9	91.1	34	1.351	8.9	58.7
	Benxi	1471	49.0	45.8	79.7	34.6	1.424	8.9	60.9
	Dalian	28191	48.4	39.5	69.2	29.9	0.897	11.6	62.5
	Dandong	9690	50.7	40.8	68.9	24.6	1.246	7.3	69.8
	Fushun	3367	50.3	47.3	84	33.3	1.132	8.9	58.7
	Fuxin	805	47.1	44	88.1	27.1	1.072	8.6	54.7
	Jinzhou	4545	50.9	54.1	85.6	37.2	1.088	10.0	49.0
	Liaoyang	3046	49.4	50.8	89.2	30.4	1.303	8.9	58.7
	Panjin	4779	48.3	43.2	71.7	28	0.952	8.9	58.5
	Shenyang	26846	49.4	57.2	96.2	40.9	0.965	8.9	58.7
	Tieling	1481	50.1	51.2	88.3	31	0.818	8.9	58.7
	Yingkou	9736	49.1	44.9	72.8	29.8	0.879	10.4	62.8
Ningxia	Yinchuan	7	54.4	47.2	107.6	34.5	1.057	9.7	47.9
Qinghai	Xining	13597	42.3	45.4	103.3	34.7	1.369	6.4	58.9
Shandong	Heze	7	51.0	81.6	143.5	38.3	1.519	15.0	66.2
	Jinan	18812	48.0	76.3	146.2	48.7	1.235	14.9	56.9
	Liaocheng	12103	49.2	85.3	149.7	41.2	1.486	15.1	62.0
	Linyi	48126	47.7	66.8	129	44.6	1.268	14.4	65.9
	Rizhao	11274	49.8	55.5	97.7	37.4	0.928	14.0	67.7

	Taian	18632	49.7	64.1	113.8	39.5	1.273	14.9	56.9
	Weifang	12230	47.7	65	125.5	35.2	0.954	14.3	62.5
	Yantai	9054	48.8	39.3	74.1	33	0.811	13.1	66.0
	Zaozhuang	6228	47.7	75.5	139.6	30.4	0.863	15.2	62.3
	Zibo	10	49.6	75.7	137.8	53.1	1.92	14.3	61.1
Shanxi	Jincheng	625	51.6	60.2	113.2	37.5	1.944	13.1	59.7
	Jinzhong	7290	48.7	60	109	36.8	1.445	11.4	54.6
	Linfen	2464	48.7	71	114.4	34.4	2.316	10.2	63.7
	Taiyuan	33509	49.1	62.2	118.9	42.9	1.457	11.4	54.6
	Yuncheng	6249	50.4	67.7	111.6	32.9	1.909	14.3	59.9
Shaanxi	Hanzhong	4163	46.1	51.3	79.4	27.1	1.301	11.9	74.9
	Xi’an	69293	45.0	67.3	132.1	50.5	1.644	15.6	59.2
Shanghai	Shanghai	2020	46.8	45.6	65	43.7	0.799	17.8	73.9
Sichuan	Chengdu	10789	47.9	57.8	96.3	48.8	1.008	16.6	81.8
	Deyang	11	48.7	52.4	88.9	28.5	0.926	16.5	82.0
	Guangyuan	460	46.4	23	59.9	31.8	0.794	16.3	69.6
	Meishan	2470	45.4	55.6	85.5	30.6	0.672	16.5	82.0
	Mianyang	10190	47.6	47.8	75	33.4	0.908	17.9	75.4
	Nanchong	4643	47.2	52.4	80.2	29.3	0.837	17.9	75.4
	Neijiang	11544	47.0	53.6	77.1	27.2	0.701	17.8	81.1
	Panzhihua	193	43.5	32	65.1	33.5	1.603	20.7	57.9
	Suining	23	44.4	44.8	75.2	23.2	0.874	17.8	81.1
	Ziyang	19603	48.1	39.2	82.6	20.4	0.738	17.8	81.1
	Zigong	29	44.1	71.3	100.3	31.5	0.883	17.7	81.7
	Luzhou	7242	49.7	58.3	84.5	29	0.573	18.7	80.3
Tianjin	Tianjin	7523	49.2	66.6	109	47.5	1.403	12.6	57.3
Xinjiang	Hami	7620	45.3	35.2	97.4	21.6	1.189	10.5	39.7
	Shihezi	14	53.1	54.6	95.4	36.7	1.184	7.8	55.9
	Urumqi	14791	46.0	72.9	128.7	50.2	1.454	3.0	59.1
Yunnan	Baoshan	1748	44.0	27.3	46.9	14.1	0.886	21.0	70.6
	Chuxiong	5209	46.1	22.1	37.9	20.1	0.809	16.5	72.9
	Dali	9270	44.2	24.2	32.7	16.1	0.624	15.5	67.0
	Kunming	25310	43.9	27.1	53.3	27.2	0.952	16.5	72.9
	Lincang	4144	44.6	26.6	42.5	15.4	0.901	19.3	74.3
	Puer	3791	45.0	25.4	45.5	15.1	0.535	19.3	76.7
	Qujing	4945	44.4	29.5	52.7	20.6	0.945	13.1	68.9
	Yuxi	9228	43.9	23.6	44.2	19.8	1.323	16.5	72.9
Zhejiang	Hangzhou	15447	45.3	47.8	75.2	42.8	0.852	18.2	73.6
	Huzhou	3977	46.5	58.8	83.7	49.7	0.949	18.2	73.6
	Jiaxing	1526	47.2	45.8	71.2	38.9	0.819	17.9	78.5
	Jinhua	20174	45.4	47.2	66	37.2	0.843	19.0	73.8
	Lishui	5243	48.1	34.4	52.1	24.9	0.687	19.6	72.8
	Ningbo	6811	46.4	39.5	63.3	39.7	0.814	18.0	78.1
	Taizhou	21355	47.2	36.5	61	22.8	0.763	18.1	79.6

	Wenzhou	3596	45.4	39.6	68.2	41.8	0.83	18.7	74.0
	Quzhou	5891	48.7	41.8	64.7	32.5	0.88	19.6	72.8
